# Improving cataract services by asking patients for their feedback

**Published:** 2022-12-16

**Authors:** Amos Kibata Githeko, Stephen Gichuhi

**Affiliations:** 1 Medical Director: CEO City Eye Hospital, Nairobi, Kenya.; 2 Senior Lecturer, Chairman: Department of Ophthalmology, University of Nairobi. Kenya.

**Having a positive experience of cataract surgery makes patients more likely to recommend the service to others. Finding out what patients think is worthwhile, as it may result in low-cost improvements that can have a significant impact.**

**Figure:**
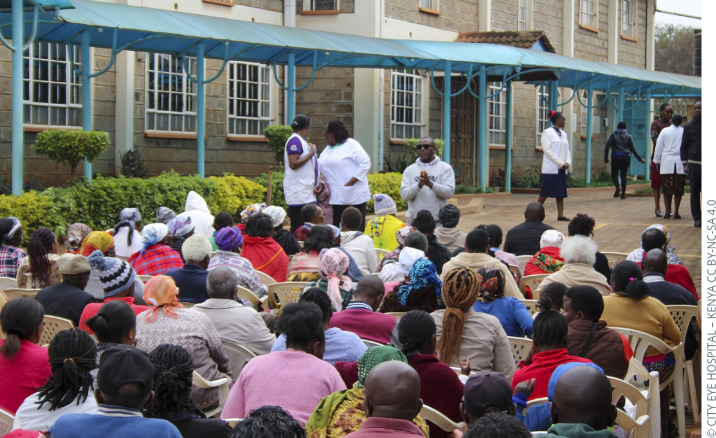
Speaking to patients outside City Eye Hospital. **KENYA**

Cataract is the leading cause of blindness globally. The VISION 2020 programme prioritised increasing the number of cataract operations performed and improving service coverage. More recently, the World Health Organization (WHO) World Report on Vision emphasised integrated people-centered eye care.^1^ Among the ten key messages of The Lancet Global Health Commission on Global Eye Health was that high quality eye health services are not always delivered.^2^

## Why does quality matter?

One of the top five challenges in eye health today is improving cataract surgery services: their quality, equity and access.^3^ WHO defines quality of care as the degree to which health services for individuals and populations increase the likelihood of desired health outcomes and describes good quality services as effective, safe, people-centered, timely, equitable, integrated, and efficient.^4^

**Figure:**
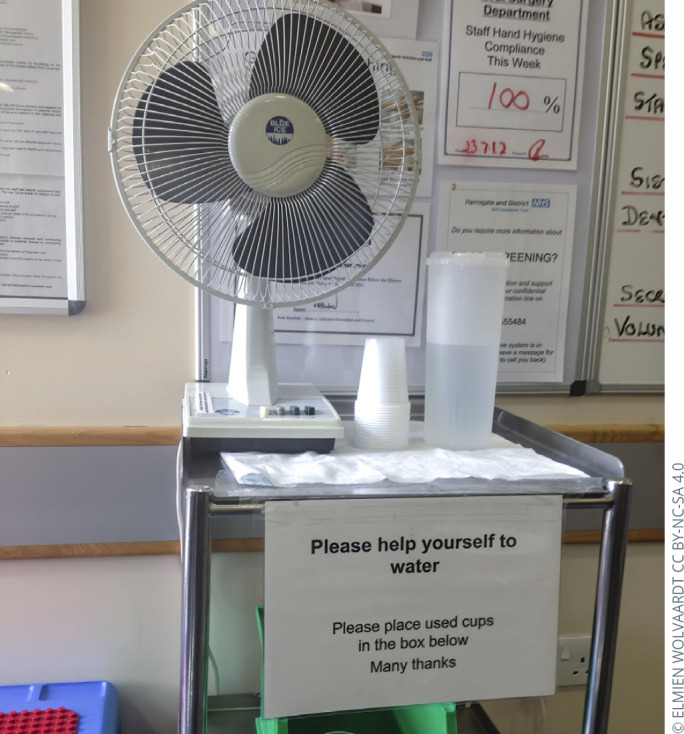
Providing drinking water is a simple way of improving patients’ experience of an eye service. **UK**

Good quality services attract more patients, which is vital for improving demand for, and uptake of, cataract services – which is important if we are to address the surgical backlog in many countries.

But how can we improve? Monitoring clinical outcomes is an important first step (see the article on page 6). If you are already doing this, the next step is to look at patients’ experience and how that can be improved.

The case study below, although imperfect, shows that speaking to patients can highlight improvements that can be made at low cost while still significantly improving patients’ experiences. Ideally such surveys should be repeated annually so that improvements can be tracked.

## Case study: Learning from our patients

City Eye Hospital is a busy day surgery centre in the city of Nairobi, Kenya that sees around 200 patients every day. Most cataract operations are done by phacoemulsification under topical anaesthesia. In 2022, we decided to find out more about our patients’ experience of the service, with the aim of finding out how our service could be improved.

Because we were short on staff time, we looked at questions researchers in other countries had asked their patients about their experience before, during and after cataract surgery^5^ and created a patient satisfaction questionnaire that we thought would be reasonably relevant in our setting. Our aim was not to produce published research, but rather to inform ourselves about how we could improve.

Over three days in June 2022, a customer service staff member asked patients waiting in different areas in the hospital whether they were willing to be interviewed. If a patient agreed, and had received cataract surgery within the previous month, the staff member asked them to rate, on a scale of 1 to 5, how satisfied they were with a set of statements about their care (see panel). The statements included aspects of care before surgery, on the day of surgery, and after surgery. A total of 62 patients completed the questionnaire over the three days.

**Questionnaire: aspects of care before, during, and after surgery**

We selected the following questions as being relevant to our patients and our service. Patients were asked to rate each item using a 5-point Likert scale:

**1** (very dissatisfied), **2** (dissatisfied)

**3** (neutral), **4** (satisfied), **5** (very satisfied)

**Before surgery**

- Easy to come to the clinic (directions)
- The waiting time before opening a file/card
- The waiting time before seeing the doctor
- Access to food and drinks while waiting
- Friendliness of staff members
- Easy access for people with disabilities
- Ease of movement for someone who cannot see well
- Privacy in the consultation room
- Doctor explained what the problem was (cataract) in a way I could understand
- Doctor took enough time with me (not rushed)
- Provided written information about cataract surgery
- Told me the cost of surgery beforehand
- Gave me a date for surgery
- Answered my questions
- Reminded me of the surgery appointment date
- Accessible by phone if I had questions

**During surgery**

- Waiting time before going into the operating room Surgical procedure was explained to me
- I found the staff helpful
- Pain during surgery

**After surgery**

- Do’s & Don’ts after surgery clearly explained
- Could see better after surgery
- Use of eye drops was clearly explained
- Gave me a number to call in case of emergency

Patients who were ‘satisfied’ or ‘very satisfied’ were graded as being happy with the service and those who were ‘unsatisfied’ or ’very unsatisfied’ were graded as being unhappy with the service.

Patients were happy about most aspects of the service, and no-one was ‘very dissatisfied’ with any aspect, which was encouraging. All were happy that they could see after surgery: 77% were very satisfied and 23% were satisfied.

However, we were keen to find out what aspects patients were less satisfied with, as that showed where we could make improvements.

The results show that the patients’ main source of dissatisfaction is not their clinical care. Patients were unsatisfied with the following:

- A lack of provision of a cataract surgery brochure before surgery that they or a family member could read (86%)
- Pain during surgery (58%)
- Lack of accessibility by phone if they had questions before surgery (54%)
- Long waiting times in the queue to open a file (22%), to see the doctor (22%) and when waiting for your turn on the day of surgery (23%).

Only pain management required a change in clinical practice. One possible solution would be to train nurses to give sub-Tenon’s blocks prior to surgery, and we are currently investigating this.

We have also addressed patients’ dissatisfaction with the absence of a contact number – we now give them a number to call if they have concerns before or after surgery. Shortening waiting times and providing written information about cataract surgery are more difficult to address, but we are looking at ways this can be done.

## Lessons for the future

Although we used a five-point scale, very few of the responses were in the middle (neutral), as we would normally expect. This suggests that having a staff member administer the questionnaire may have influenced patients’ responses. For example, patients may have been worried that a negative response could influence the care they receive in future. We could improve on this next time by asking someone to help who is independent of the hospital, and is perceived as being independent, to administer it.

Another limitation of our approach is that we chose the questions based on what we thought was important at the time, which might not reflect all of the concerns our patients have. It is possible we could have addressed this by adding an open-ended question at the end, to find out what else patients think we should have asked about. In future, we could also ask someone experienced in qualitative research to speak to smaller groups of patients first, to find out what is important to them, and then use the results when drawing up the questionnaire.

Although our results cannot be generalised to other clinics, or used to compare the patient satisfaction in this eye hospital with the results from other eye units, we plan to repeat key questions in 12 months’ time to check whether the changes we made have led to better patient satisfaction.

**Previous articles**

**How Aravind Eye Care System assessed patient satisfaction and improved uptake of eye care by 15% by providing a better patient experience**

**https://www.cehjournal.org/article/patients-perspective-an-important-factor-in-assessing-patient-satisfaction/**

**Practical tips on how to provide a positive patient experience:**

**https://www.cehjournal.org/article/improving-the-patients-experience/**

**Different ways to find out what patients think about our services & what the challenges are (including a great case study from KCMC):**

**https://www.cehjournal.org/article/understanding-what-patients-think-about-eye-care-and-our-services/**
